# Systemic Treatment by Dentists

**Published:** 1896-03

**Authors:** 


### Editorial, Etc.

**SYSTEMIC TREATMENT BY DENTISTS.**—The physician and the dentist have necessarily many practical duties in common, but each has a clearly defined limitation of sphere requiring specific direction of that general culture which both should possess. It is not the purpose in any thing that is said to advise the dentist to usurp the office of the physician, whose therapeutics is restricted to hygiene and materia medica; but it cannot be denied that disease of the body involves an unhealthy condition of the mouth. Those best qualified to judge, the men endeavoring to raise dentistry to the sphere it should occupy, have always deemed a full medical and surgical education desirable by practitioners devoting themselves to dentistry. Oculists and aurists and obstetricians or other physicians or surgeons restricting themselves to or selecting one branch of practice in preference to another are at the same time fully-qualified medical men; and as medical men they have objected to what they term "fractionally-qualified" being made to appear on the equal footing with themselves. Nevertheless it must not be forgotten that the dentist often waits upon patients who need systemic treatment more than treatment of the teeth, and the first effort in the treatment of caries should be directed to the predisposing and exciting causes, constitutional as well as local. The teeth are a part of the animal economy and they are therefore more or less influenced by the state of the general health. In many persons, especially those who have become run down, there is more or less nervous irritation, often without any perceptible disturbance in the teeth themselves which very decidedly affects the health in one way or another. The connection of the teeth with various constitutional diseases should not be overlooked.

What Dr. Louis Jack observes respecting the uncertainty of dental service, should be persistently kept before the minds of patients. He says: "In some instances comparatively indifferent operations are successful, and in other cases those performed with the greatest care have proven failures. In the former correct physiological function may have become re-established, and in the latter, either from defective hygienic conditions or from chronic impairment of function, the active causes of carious action have continued. Hence the failures of operations on the teeth is not always an evidence of deficient skill."

The dentist is too apt to confine himself to his branch of study and to ignore the human system as a whole. He should consider other things besides enamel and dentine and pulp. The educated dentist should by scientific, therapeutic and surgical remedies as well as by mechanical applications combat presenting conditions. Says Dr. A. O. Hunt, in discussing the softening about the margins of fillings:

"Almost any of the disturbances of the liver, kidneys, lungs, etc. would cause a change in the secretions of the mouth. One case which occurred to him at the moment was that of a gentleman, 55 or 60 years of age, in which this softening around fillings in good teeth was observed. A portion of the decay was removed and sent over to the laboratory for examination. The report of the examiner was that the bacteria of pulmonary phthisis was present in large numbers, for which he was not looking, although he did expect evidence of some constitutional difficulty, as diabetes or Bright's disease."

It seems consistent with the ethics of the profession to note that a general and nerve tonic may be of value if prescribed in dental practice. R. G.

